# Supplementary material for: “A custome lothsome”: Investigating the association between tobacco consumption and respiratory inflammation in two post-medieval English populations (c. CE 1500–1855)
Source: PLoS One. 2025 May 22;20(5):e0324045. doi: 10.1371/journal.pone.0324045 (PMC12097713; doi:10.1371/journal.pone.0324045)
Supplement: Attachment — Submitted filename: Review_PONE-D-24-53580.docx [file pone.0324045.s002.docx]

“A custome lothsome”: Investigating the association between tobacco consumption and respiratory inflammation in two post-medieval English populations (c. CE 1500–1855)

Manuscript number: PONE-D-24-53580

The topic of the paper manuscript is interesting and as stated the effect of tobacco on health in past populations is not well understood, so the paper rightfully offers to lay a steppingstone towards understanding this. However, I find that the paper too comprehensive not sticking to the major focus (see comments) and I think will benefit greatly from restructuring and taking out parts of analysis. I have some concerns and general and more specific recommendations for changes:

**General comments**

First, the data used is not ideal for the analysis made and conclusions that is sought drawn from them. It is a problem that no medieval ‘baseline’ sample is included for the urban site and the baseline sample from the rural site is very small (especially when grouped into sex and age groups). Without also including a urban 'baseline' I would exclude the medieval 'baseline', - it does not really bring much information and is not used actively in overall conclusions. Furthermore, the post-medieval rural population covers the time period 1500 – 1855 which is very wide and thus not very comparable to the chronologically narrow urban post-medieval population dated within 1789-1853. If possible to group the chronology of the rural site into early and late post-medieval period it would be very beneficial to the comparative analysis of the two sites. Furthermore, in general the samples used are as pointed out by the authors very small when divided into sex, age groups, non-smoker/smoker, social status, chronological groups and urban/rural and therefore not much can actually be concluded.

Regarding the methods for assessing smoking status. Have you considered whether levelling how much you smoke would be important for the results of the study and also could the degree of smoking be divided into levels using the applied methods for distinguishing smokers from non-smokers. A mention of this either in regards to methods or in the discussion would be an interesting addition to the perspectives of the research topic.

Also, it is stated (lines 12-14) that; *the paper will explore the potential respiratory health implications of the rapid incorporation of tobacco-use into the everyday lives of English citizens during the post-medieval period* but a study of the general prevalence of the respiratory diseases without looking at smoking status is also included. This results in a presentation of results and discussion that is very confusing to read and leaving the reader unsure about what exactly is being discussed and whether results, discussion or conclusions are minded on non-consumers vs. consumers or just generally on SES groups, dating groups or rural/urban. This is in my opinion something not directly related to the purpose of the paper and the paper would benefit from leaving the prevalence of the respiratory diseases out when not studied in relation to non-smokers and smokers. This would also contribute to solving some of the problems with small samples size because in the present version the small groups and many different potential confounding factors sought explored makes the conclusions very weak, and in many instances not reliable as no statistical test results to back them up. I think the authors in general take conclusions too far without stats to support them.

I think much more emphasis is needed on discussing tuberculosis as a major confounding factor for the respiratory disease indicators especially indicators of pulmonary infection. This disease is known as highly prevalent especially from late 18^th^ century and in the analysis that shows no differences among smokers and non-smokers an explanations could be that many where infected with tuberculosis with active disease affecting the respiratory system especially the longs resulting in periosteal reaction on visceral surface of ribs. Tuberculosis is only mentioned shortly in line 338 in connection to transmission from wastewater.

And also too little discussion and emphasis on interpretation of results in the light of the osteological paradox in regards to the prevalence of bony indicators of both maxillar sinus infection and pulmonary infection. Both conditions (or implications from co-infection) could very likely cause death before bone involvement which will have a major impact on the possibilities to draw the conclusions made. This is mentioned briefly in line 391 in relation to lower-class smoking women that did not survive respiratory insults long enough to form evidence of inflammation, but this deserves a section on its own.

Tables 1 and 2: No statistical tests of the differences in prevalence rates?

Figure 5: Confusing, - gives only prevalence rates and no counts or statistical test results therefore difficult to assess the significance of the distributions shown. Need to turn to large tables 1 and 2 for counts which is confusion for the reader. I think the figure is misleading because the lack of significance of the differences of the distributions cannot be seen in the figure.

**Specific comments**

Line 14: Adult skeletons

Line 16: assessed not assed

Lines 68, 69, 70: Referencing should be listed in end notes as for the remaining manuscript

Lines 72 – 75: will the absence of pipe-notches and staining on dentition be evidence of non-smoking?

Lines 89-93: Very long and a bit unclear - consider rewriting and splitting up in two sentences.

Lines 99-100: If not tested against smoking status how does this give insights into the effect of tobacco smoking on the health in the past?

Line 100: (see 37) should be changed to (37).

Line 115: what date/definition of post-medieval period are you using?

Line 125-126: 62 skeletons (note the sheet with raw data provided has 60 medieval skeletons listed) that later are split into males and females, is a very small sample for providing a ‘baseline’ for pre-tobacco respiratory disease prevalence and a problem that this ‘baseline’ sample only is provided for the rural population because later rural and urban differences are discussed.

Line 156-157: (see 4) changed to (4) or identified according to the methods for registration presented by Davies-Barret and Inskip (4). How certain will a non-smoking status be without any of these indicators?

Line 157ff: it is mentioned that when not possible to identify tobacco consumption status from osteoarchaeological methods biomolecular methods were applied – why were biomolecular methods not applied to all?? When data about smoking status not collected in the same manner what implications for final results?? Please write in main text how many with tobacco consumption status assessed from dentition and how many from biomolecular analysis.

Lines 166ff: I know these are standard methods and widely used, but it is unfortunate that old and not very accurate methods for aging are used when newer methods are available – ex. the latest version of transition analysis (<https://www.statsmachine.net/software/TA3/>).

Line 169: Also not very ideal way of grouping (but know it is used as a standard) here the age group 50+ is rather useless when some individuals in the samples likely where much older (70, 80 or 90) when they died.

Line 177: miss be between can and found.

Lines 178-180: and what were criteria for scoring? Whenever pathology present or certain amount?

Lines 230: Statistical significance? And what table to look for results?

Lines 263-264: compared to non-consumers?

Lines 263ff: Results presented have no statistical test results and conclusions cannot be made from the mentioned rates. Also, rates presented are a mix of individuals where no information about tobacco consumption status and with information. This is very confusing and as mentioned before I find that parts where no information about smoking status is present not really are relevant to include because they are beyond the purpose of the paper, - the results and later discussion should be kept within the purpose and aims of the paper.

Lines 272ff: Again results for groups without information about smoking status is included and do not add to insights into the relation of tobacco consumption and respiratory diseases.

Line 274: 2.8 should be changed to 2.7 (according to table 4)

Lines 314-315: non-smoking males of higher socio-economic status most affected by respiratory inflammation – a very likely explanation is that they survived longer than their smoker counterparts and thus lived long enough for bone changes related to the inflammatory diseases to develop and therefore not necessarily had a higher prevalence of disease.

Lines 315-316: Here I believe a very important confounding factor is tuberculosis infection and this deserved a mentioning and also elaboration.

Lines 321: Unfortunately the problem with the sampling makes it very difficult to test either of these potential effects in detail.

Line 323ff: Again tuberculosis needs to be mentioned.

Sections: line 355ff, line 371ff, line 382ff and line 393ff, and line 407ff: have many parts in which discussion of respiratory disease in general is made but without the perspectives of tobacco consumption. In my opinion when not really linked to results of the paper not involving tobacco consumption this is not relevant for the current paper.

Lines 432-434: Or the fact that general health improves and life expectancy increase and people live longer with respiratory disease and develop more bone changes.

Lines 456-460: therefore suggest leaving out some of these potential factors to get more clear picture of the impact of tobacco consumption.

Line 488-489: Or they lived long enough to develop bone changes.
