# Supplementary material for: “A custome lothsome”: Investigating the association between tobacco consumption and respiratory inflammation in two post-medieval English populations (c. CE 1500–1855)
Source: PLoS One. 2025 May 22;20(5):e0324045. doi: 10.1371/journal.pone.0324045 (PMC12097713; doi:10.1371/journal.pone.0324045)
Supplement: Attachment — Submitted filename: PONE-D-24-53580 Review.docx [file pone.0324045.s003.docx]

The submitted paper presents an application of biochemical data to palaeopathological explorations of respiratory health with consideration of whether tobacco consumption affects the prevalence of osteological evidence for respiratory disease. The results are interesting and highlight the complexities of identifying and adequately accounting for increasing environmental risk factors for disease that occurred with intensive industrialisation and urbanisation and how these can mask other factors that may present clearer data in other settings.

A number of comments are presented below which I think would improve the presented work in its clarity for readers.

Abstract

1- Page 1, Line 16: Typo identified – original text “Individuals were assed”. Assume this is meant to say, “Individuals were assessed”.

Main Text

2- Page 2, Line 44: Original text “While tobacco-use occurred frequently across all strata of society, the way people consumed became”. Suggested change “the way people consumed tobacco”. The sentence will read easier with it being explicitly being stated what people were consuming.
3- Page 3, Lines 54 – 62: Original text: “It induces respiratory inflammation, damages the tissues of the respiratory tract ….. oral diseases (23-26), to name but a few conditions associated with consumption of the drug.” This section has a repetitive feel to it due to the first two sentences both stating that the consumption of tobacco will increase infections/infectious diseases.

The opening sentence of this section of text could be improved by either a) removing the reference to infection (“promotes infection”) or b) moving this towards the end of the sentence and mentioning non-infectious disease too (“tract, increasing susceptibility to a range of infectious and non-infectious respiratory diseases”.

The start of the second sentence should also be reworked to remove “increases the risk of” portion as this will help reduce the sense of repetitiveness that currently exists across the first three sentences (lots of use of “increase”/”increased”).

4- Page 4, Line 76: Original Text “Inskip and colleagues (32). There is inconsistent use of “and colleagues” versus “et al.” (used on line 79) through the paper.
5- Page 6, Figure 2, Caption: The reference for this figure (Davies-Barrett et al., in review) is not listed in the references at the end of the paper.
6- Page 6, Line 139: Original text “‘rate payers’ (those who owned property)”. Please define rate payers in line 134 instead as that is the first use of the term in the paper.
7- Page 9, Lines 209 to 212: Original text “In binary logistic regression, if the upper limit of the 95% confidence interval falls below one”. The use of “one” (in text form) in this sentence and the sentence explaining the lower limit is unclear. Does the text mean that the 95% confidence intervals between groups are compared, or does the text mean that the limit of the falls above/below the numerical value of 1? If it is the latter, “one” should be written as “1” so it is clear that a numerical value is referred to here.
8- Page 9, Line 217 and Figure 4: Original text “However, a lower frequency of mature adults”. Frequency refers to the rate of something in a period of time or sample and so should not be used to refer to a number which is not a rate. Please change the use of “frequency” in this line of text to “number” and to “Number of individuals” on the y-axis of the figure.
9- Page 14, Figure 5: This is a really interesting way of visually representing the collective results from the study. I wonder whether numerical labels on the circles would help with the readability of the figure e.g. if 0°-180° is vertical with 0° at the top of the circle, 0° would have 0%, 90° and 270° would have 50% and 180° (bottom of the circle) would have 100%).
10- Page 20, Line 408: Original text “(although not this result was)”. Assumed typo and “not” is meant to be “note”.
