# Supplementary material for: “A custome lothsome”: Investigating the association between tobacco consumption and respiratory inflammation in two post-medieval English populations (c. CE 1500–1855)
Source: PLoS One. 2025 May 22;20(5):e0324045. doi: 10.1371/journal.pone.0324045 (PMC12097713; doi:10.1371/journal.pone.0324045)
Supplement: Attachment — Submitted filename: Responses to Reviewers.docx [file pone.0324045.s005.docx]

**Author’s Responses to Reviewers’ Comments**

Dear Editors and Reviewers. We thank you for taking the time to make comments on our manuscript, which have been very useful in improving the quality of our paper. We have outlined our responses to your comments below; major changes include expansion on the topics of tuberculosis and heterogenous frailty, clarification of methods, and the inclusion of a limitations and future directions section within the discussion to more directly address some of the concerns raised.

**Journal requirements:**

When submitting your revision, we need you to address these additional requirements.

1. Please ensure that your manuscript meets PLOS ONE's style requirements, including those for file naming. The PLOS ONE style templates can be found at

https://journals.plos.org/plosone/s/file?id=wjVg/PLOSOne_formatting_sample_main_body.pdf and

<https://journals.plos.org/plosone/s/file?id=ba62/PLOSOne_formatting_sample_title_authors_affiliations.pdf>.

The manuscript has been adjusted to conform to PLOS ONE style requirements.

2. Please amend either the title on the online submission form (via Edit Submission) or the title in the manuscript so that they are identical.

The two titles are already identical.

3. In your manuscript, please provide additional information regarding the specimens used in your study. Ensure that you have reported human remain specimen numbers and complete repository information, including museum name and geographic location.

If permits were required, please ensure that you have provided details for all permits that were obtained, including the full name of the issuing authority, and add the following statement:

'All necessary permits were obtained for the described study, which complied with all relevant regulations.'

If no permits were required, please include the following statement:

'No permits were required for the described study, which complied with all relevant regulations.'

For more information on PLOS ONE's requirements for paleontology and archeology research, see https://journals.plos.org/plosone/s/submission-guidelines#loc-paleontology-and-archaeology-research.

All skeletal identifier numbers are given in Supplemental File S1 (raw data). Storage information at time of analysis has been given. Neither skeletal collection included in this study belonged to a museum and these collections have been moved since analysis. The information of their current location is no longer available to the authors. The phrase “No permits were required for the described study, which complied with all relevant regulations” has been inserted into the main body of the text.

4. We note that the grant information you provided in the ‘Funding Information’ and ‘Financial Disclosure’ sections do not match.

When you resubmit, please ensure that you provide the correct grant numbers for the awards you received for your study in the ‘Funding Information’ section.

Funding information has been amended in the manuscript. The grant numbers remain correct.

5. Thank you for stating the following financial disclosure:

[This research was mainly funded by a UKRI-FLF research grant (grant no.: MR/T022302/1), awarded to SI. Additional funding came from a Dutch Research Council (NWO) grant (grant no.: PGW.21.008), awarded to MC.].

Please state what role the funders took in the study. If the funders had no role, please state: ""The funders had no role in study design, data collection and analysis, decision to publish, or preparation of the manuscript.""

If this statement is not correct you must amend it as needed.

Please include this amended Role of Funder statement in your cover letter; we will change the online submission form on your behalf.

The above statement has been added to the financial disclosure in the manuscript.

6. Your ethics statement should only appear in the Methods section of your manuscript. If your ethics statement is written in any section besides the Methods, please move it to the Methods section and delete it from any other section. Please ensure that your ethics statement is included in your manuscript, as the ethics statement entered into the online submission form will not be published alongside your manuscript.

The ethics statement has been moved to the main body of the text.

7. We are unable to open your Supporting Information file [Supplemental File S1 - Raw data.sav]. Please kindly revise as necessary and re-upload.

This file has been converted to a CSV file for better accessibility and reuploaded.

**Comments to the Author**

**Reviewer #1: Dear authors,**
this is a very well-written manuscript and well-planned research. I appreciate the opportunity given to read your manuscript. I have only minor observations that the authors should consider, they mostly express a concern with the need to avoid over simplification views of what can be inferred based on the analysis of human remains

1. For example, in line 52 “Today, smoking is synonymous with concepts of blackened, diseased lungs and poorly, wheezing individuals.” - this is a very general description, please provide more detail, or be more specific. Throughout the manuscript, often, some arguments offer a very generalist view of the facts - this should be avoided.

This statement has been removed from the text.

2. in Lines 71 - 83: although adequate, this section ignores the relevance of differential diagnosis when exploring bone changes. The authors provide an argument that focuses on the relation between smoke and disease-related bone changes, failing to address the complexity of disease-related bone changes etiology.
   As the authors must certainly know the presence of high levels of prevalence and/or high correlation is not necessarily a synonymy of causation. This needs to be brought to the Introduction and discussion.

In these lines we present only the findings from previous bioarchaeological studies on the topic. We do not present here any arguments of our own. Are you referring to a different section of text? Regardless, we understand the importance of addressing the complexity of disease-related bone changes and their aetiology and this has been further expanded upon in a new limitations section.

3. Also, please state if you have permission to reproduce figure 1, even it being an adaptation, this needs to be clear.

This image is available under a CC-BY 4.0 licence and a statement to this effect has been added to the caption.

**Reviewer #2:** The manuscript presents new data and important new insights into respiratory health and the impact of tobacco consumption in the past. The statistical tests applied are appropriate for the sample based on the kind of data collected and the size of the cohorts, and the tables and figures are clear and well presented.

1. There are a few points which the authors may wish to elaborate; a sentence or two giving a working definition of intersectionality as it is understood in biological anthropology would be very useful at the outset.

This has been added to the introduction.

2. Following this, for example in lines 380ff, it would be interesting to explore these data in light of the framework for intersectionality you have presented; only a line or two about what these confounders might be would suffice (which you have done with discussion of other cohorts).

This has been expanded upon in the discussion.

3. The following is a line-by-line review of some typos and stylistic edits that could be applied.
   ll. 16 : assessed
   ll. 21: over two times - could become : more than twice as likely
   ll. 28: delete comma after of
   ll. 59: remove commas
   ll. 66: replace : to the risks, with, of the risks
   ll. 67: delete against (this is already implied in the meaning of the word decry)
   ll. 122ff: ensure that there is a space after the date and before CE
   ll. 176: suggest starting this line with, A discussion of the pathophysiological processes...
   ll. 203-204 : italicise p in p-value
   ll. 206: introduce the abbreviation of odds ratio (OR)
   ll. 371: The data also hints at...(to avoid use of the passive voice here)
   ll. 184 and 510: The full trading name of Karl Storz should be represented here: Karl Storz SE & Co. KG, and perhaps city and country if required by journal submission guidelines.

All amendments have been made.

**Reviewer #3**: The present work is an interesting attempt to analyze the impact of smoking on human health using osteoarchaeological and metabolite analyses of English citizens during the post-medieval period. Overall, the paper is well-prepared; however, in my opinion, two issues are not fully addressed and require further consideration:

1. Sample size: The study relies on a relatively small sample size, especially when the examined groups are further subdivided based on multiple variables (tobacco consumption, age, sex, and socioeconomic status). This issue is particularly pronounced for the Barton-upon-Humber site, where subdividing the sample results in very small groups (N<10; see Tables 1 and 2). As a result, the ability to draw reliable conclusions from the data is limited. Did the authors consider including additional samples, particularly from the pre-tobacco period, to increase the sample size?

Unfortunately, this common pitfall in bioarchaeological studies was unavoidable in the current study. We assessed all available post-medieval individuals from the Barton-upon-Humber cemetery for which we had secure dating. Issues with precise dating of the site have meant that, despite perceived large numbers of individuals, we could not expand our sample for the post-medieval period. Data for the medieval period within the Barton-upon-Humber cemetery was collected from a reduced sample as a control and unfortunately time and funding did not allow for a greater sample size. A medieval sample associated with the cemetery of St James’s, London, was unfortunately unavailable and the addition of another medieval cemetery from a different part of London would have introduced additional variables. Further, final sample sizes have been reduced by the inability to always accurately identify sex, age, and smoking status. We have expanded upon this in a new limitations section.

2. Controlling for confounding factors: The authors’ assumption that maxillary sinusitis and pulmonary/pleural inflammation can be directly linked to tobacco use, despite the multifactorial etiology of these changes, is questionable. While the authors acknowledge this issue in the Discussion section and suggest it as a reason for the ambiguous results, it remains unclear how they intended to control for confounding factors (if this is even possible, which I sincerely doubt) and demonstrate that the observed respiratory diseases were specifically caused by tobacco smoking rather than other factors.
   We only hypothesise that respiratory inflammation can be linked to tobacco consumption. Our research questions are aimed at exploring a statistical association between tobacco consumption and respiratory inflammation but we clearly state that tobacco will not be the only cause for respiratory disease. We also statistically assess the implication of other factors on respiratory disease on these populations and discuss the outcomes and likely influence of confounding factors extensively in the discussion. As we state in the discussion and conclusion, there are a number of causes for respiratory disease that complicate a potential association and it is likely that confounding factors worked in tandem to affect susceptibility to respiratory disease. Nowhere do we definitively state that the respiratory disease present in our populations is solely caused by tobacco consumption, we only suggest that tobacco consumption is a likely contributing factor that has thus far largely been ignored by other post-medieval studies of health. We have expanded upon the different aetiologies of the bone changes examined in this study in a new limitations section.

Other comments:

3. The Material section lacks information on the percentage of individuals excluded from the study due to the lack of suitable skeletal elements or the poor state of preservation of the human skeletal remains. Please include this information.

We have added this information to the methods section.

4. The Methods section lacks a brief description of how the molecular analysis was performed. Simply stating that "metabolomic identification of small molecules (metabolites)" was used is insufficient, especially since this method is not commonly employed in osteoarchaeological research. While citations for the methods are provided in the Introduction, they are absent from the Methods section, except for those concerning the signatures used. Additionally, there is no information regarding the research laboratory protocol followed during the analysis. Were all steps carried out as described in the paper by Badillo-Sanchez et al. (2024)? For what percentage of all individuals was metabolite analysis performed? Were the analyses successful for all these individuals?

We have expanded upon this in the methods section.

5. It is important to emphasize that exposure to tobacco smoke, and not just active smoking, induces changes in the sinuses and ribs. How can second-hand smokers be distinguished in osteoarchaeological studies? If such identification is not possible, how can it be ensured that the authors have correctly classified individuals exposed to tobacco smoke versus those who were not? This issue is particularly significant, especially since "it is yet unknown whether tobacco inhalation via second-hand smoke (…) creates a metabolic signature similar to direct smokers". Please clarify.

We agree this is an important issue and we have expanded upon this in our methods and limitations sections.

6. How were data on individuals classified as "possible" and "probable" women or men interpreted? Were they included in the results as women/men, even though the assessment was uncertain? If so, could this have contributed to the ambiguous results? Additionally, which numbers in the sex groups in the supplement correspond to these categories? This has not been clearly explained in the text.

Greater clarity on this has been provided within the text. The supplemental information has been reformatted into a CSV file for greater clarity and accessibility.

7. The authors provide an insightful overview of the importance of tobacco in post-medieval England. However, the manuscript does not address whether regional variations in tobacco use existed during this period. Is this information available?

Unfortunately, direct information on this is generally unavailable or very hard to find, and currently beyond the scope of this study.

8. Abstract: The word "assed" should be corrected to "assessed."

This has been corrected.

9. Discussion: The sentence "Some intersectional impacts are also hinted at." lacks clarity and specificity.

This sentence has been adjusted

10. Figure 1: Map lacks a scale, making it difficult to accurately assess the distance between sites.

A scale has been added.

11. Figure 4: Does the y-axis represent the frequency or indicate the number of individuals within each category?

The labelling on this figure has been adjusted.

**Reviewer #4:** The topic of the paper manuscript is interesting and as stated the effect of tobacco on health in past populations is not well understood, so the paper rightfully offers to lay a steppingstone towards understanding this. However, I find that the paper too comprehensive not sticking to the major focus (see comments) and I think will benefit greatly from restructuring and taking out parts of analysis. I have some concerns and general and more specific recommendations for changes:

General comments

1. First, the data used is not ideal for the analysis made and conclusions that is sought drawn from them. It is a problem that no medieval ‘baseline’ sample is included for the urban site and the baseline sample from the rural site is very small (especially when grouped into sex and age groups). Without also including a urban 'baseline' I would exclude the medieval 'baseline', - it does not really bring much information and is not used actively in overall conclusions.

Please see our response to Reviewer 3, Comment 1 on the subject of sample size. We would prefer to include the baseline as it provides a comparison to our post-medieval populations and could be complemented by further data in, or contribute to, future studies.

2. Furthermore, the post-medieval rural population covers the time period 1500 – 1855 which is very wide and thus not very comparable to the chronologically narrow urban post-medieval population dated within 1789-1853. If possible to group the chronology of the rural site into early and late post-medieval period it would be very beneficial to the comparative analysis of the two sites.

Unfortunately, it was not possible to provide more narrow date ranges for the rural site due to problems with the accuracy of the phasing of the site and a lack of radiocarbon dating. This would have also reduced the sample sizes even further.

3. Furthermore, in general the samples used are as pointed out by the authors very small when divided into sex, age groups, non-smoker/smoker, social status, chronological groups and urban/rural and therefore not much can actually be concluded.

This is a common problem in bioarchaeological studies, yet a breakdown such as this is essential in exploring confounding factors that may have affected respiratory health alongside tobacco consumption. We are clear in the limitations of our study on this matter within a new limitations section.

4. Regarding the methods for assessing smoking status. Have you considered whether levelling how much you smoke would be important for the results of the study and also could the degree of smoking be divided into levels using the applied methods for distinguishing smokers from non-smokers. A mention of this either in regards to methods or in the discussion would be an interesting addition to the perspectives of the research topic.

Unfortunately, with the methods currently available to us for identification of tobacco consumption, this is not yet possible. We have added expanded on this in the new limitations section.

5. Also, it is stated (lines 12-14) that; the paper will explore the potential respiratory health implications of the rapid incorporation of tobacco-use into the everyday lives of English citizens during the post-medieval period but a study of the general prevalence of the respiratory diseases without looking at smoking status is also included. This results in a presentation of results and discussion that is very confusing to read and leaving the reader unsure about what exactly is being discussed and whether results, discussion or conclusions are minded on non-consumers vs. consumers or just generally on SES groups, dating groups or rural/urban. This is in my opinion something not directly related to the purpose of the paper and the paper would benefit from leaving the prevalence of the respiratory diseases out when not studied in relation to non-smokers and smokers. This would also contribute to solving some of the problems with small samples size because in the present version the small groups and many different potential confounding factors sought explored makes the conclusions very weak, and in many instances not reliable as no statistical test results to back them up. I think the authors in general take conclusions too far without stats to support them.

It is vital to explore the potential confounding factors outside of tobacco consumption that may have affected respiratory health, in order to contextualise our findings and discuss the complexity of factors that contribute to respiratory disease. Further, a study such as this presents the opportunity to discuss respiratory diseases in the past within a wider context. We would be doing an injustice to the time and effort gone to collect the data and to the individuals we have studied, if we did not present a comprehensive analysis of the results. We are very clear in our conclusions about the limitations of our study and that further work is required on this topic.

6. I think much more emphasis is needed on discussing tuberculosis as a major confounding factor for the respiratory disease indicators especially indicators of pulmonary infection. This disease is known as highly prevalent especially from late 18th century and in the analysis that shows no differences among smokers and non-smokers an explanations could be that many where infected with tuberculosis with active disease affecting the respiratory system especially the longs resulting in periosteal reaction on visceral surface of ribs. Tuberculosis is only mentioned shortly in line 338 in connection to transmission from wastewater.

We have expanded on the implications of tuberculosis within the discussion. It should be noted however, there is a significant correlation between tobacco consumption and TB prevalence in modern clinical studies. Therefore, we cannot present tuberculosis as an accurate reason for similar prevalence rates among consumers and non-consumers.

7. And also too little discussion and emphasis on interpretation of results in the light of the osteological paradox in regards to the prevalence of bony indicators of both maxillar sinus infection and pulmonary infection. Both conditions (or implications from co-infection) could very likely cause death before bone involvement which will have a major impact on the possibilities to draw the conclusions made. This is mentioned briefly in line 391 in relation to lower-class smoking women that did not survive respiratory insults long enough to form evidence of inflammation, but this deserves a section on its own.

Maxillary sinusitis does not lead to death except in the very rarest of complications and, thus, the osteological paradox is inapplicable in this instance. We have expanded upon the osteological paradox in relation to pulmonary inflammation within the limitations.

8. Tables 1 and 2: No statistical tests of the differences in prevalence rates?

Statistical tests are presented in tables 3 and 4. It is a common approach in statistical analysis that statistical tests should be kept to a minimum to only answer direct research questions as the over-use of statistical tests decreases the significance of the outcome by introducing greater likelihood of type I error.

9. Figure 5: Confusing, - gives only prevalence rates and no counts or statistical test results therefore difficult to assess the significance of the distributions shown. Need to turn to large tables 1 and 2 for counts which is confusion for the reader. I think the figure is misleading because the lack of significance of the differences of the distributions cannot be seen in the figure.

This figure is simply to provide a visual representation of data presented in tables 1 and 2 to give an overview of prevalence rates found in this study. The figure has been adjusted for greater clarity as per suggestion by Reviewer 5.

Specific comments

10. Line 14: Adult skeletons

This has been added.

11. Line 16: assessed not assed

This has been corrected.

12. Lines 68, 69, 70: Referencing should be listed in end notes as for the remaining manuscript

This has been corrected.

13. Lines 72 – 75: will the absence of pipe-notches and staining on dentition be evidence of non-smoking?

This has been clarified in the methods section.

14. Lines 89-93: Very long and a bit unclear - consider rewriting and splitting up in two sentences.

This has been clarified.

15. Lines 99-100: If not tested against smoking status how does this give insights into the effect of tobacco smoking on the health in the past?

This point is placed within the context of tobacco-use in the text shortly below this sentence in the form of hypothesis 2.

16. Line 100: (see 37) should be changed to (37).

This has been adjusted.

17. Line 115: what date/definition of post-medieval period are you using?

Dates have been added.

18. Line 125-126: 62 skeletons (note the sheet with raw data provided has 60 medieval skeletons listed) that later are split into males and females, is a very small sample for providing a ‘baseline’ for pre-tobacco respiratory disease prevalence and a problem that this ‘baseline’ sample only is provided for the rural population because later rural and urban differences are discussed.

The discrepancy in numbers is due to the exclusion of two individuals who were too incomplete to be assessed for respiratory disease. This has been clarified in the methods section. Please see our response to Reviewer 3, comment 1, regarding sample size.

19. Line 156-157: (see 4) changed to (4) or identified according to the methods for registration presented by Davies-Barret and Inskip (4). How certain will a non-smoking status be without any of these indicators?

This section has been clarified.

20. Line 157ff: it is mentioned that when not possible to identify tobacco consumption status from osteoarchaeological methods biomolecular methods were applied – why were biomolecular methods not applied to all?? When data about smoking status not collected in the same manner what implications for final results?? Please write in main text how many with tobacco consumption status assessed from dentition and how many from biomolecular analysis.

This section has been clarified.

21. Lines 166ff: I know these are standard methods and widely used, but it is unfortunate that old and not very accurate methods for aging are used when newer methods are available – ex. the latest version of transition analysis (<https://www.statsmachine.net/software/TA3/>).

Unfortunately, transitional analysis takes a long time and was, therefore, incompatible with the time restraints of data-collection in this instance. Additionally, in the UK, we lack formal training in the use of transitional analysis and, furthermore, its accuracy has not yet been tested on British populations. These methods are not yet widely used. Transitional analysis will, however, be considered in future studies if formal training becomes available.

22. Line 169: Also not very ideal way of grouping (but know it is used as a standard) here the age group 50+ is rather useless when some individuals in the samples likely where much older (70, 80 or 90) when they died.

As you state, this is a standard approach to grouping individuals in bioarchaeological studies due to the error introduced in age estimation methods when applied to individuals from later adulthood.

23. Line 177: miss be between can and found.

This has been corrected.

24. Lines 178-180: and what were criteria for scoring? Whenever pathology present or certain amount?

This has been clarified in the methods section.

25. Lines 230: Statistical significance? And what table to look for results?

Statistical significance is discussed in the following sentences.

26. Lines 263-264: compared to non-consumers?

This has been clarified.

27. Lines 263ff: Results presented have no statistical test results and conclusions cannot be made from the mentioned rates. Also, rates presented are a mix of individuals where no information about tobacco consumption status and with information. This is very confusing and as mentioned before I find that parts where no information about smoking status is present not really are relevant to include because they are beyond the purpose of the paper, - the results and later discussion should be kept within the purpose and aims of the paper.

Please see response to comments 5 and 8.

28. Lines 272ff: Again results for groups without information about smoking status is included and do not add to insights into the relation of tobacco consumption and respiratory diseases.

Please see response to comment 5.

29. Line 274: 2.8 should be changed to 2.7 (according to table 4)

This has been adjusted.

30. Lines 314-315: non-smoking males of higher socio-economic status most affected by respiratory inflammation – a very likely explanation is that they survived longer than their smoker counterparts and thus lived long enough for bone changes related to the inflammatory diseases to develop and therefore not necessarily had a higher prevalence of disease.

This sentence has been adjusted and reflection on the osteological paradox has been expanded upon within the limitations.

31. Lines 315-316: Here I believe a very important confounding factor is tuberculosis infection and this deserved a mentioning and also elaboration.

Please see response to comment 6.

32. Lines 321: Unfortunately the problem with the sampling makes it very difficult to test either of these potential effects in detail.

Yes, we agree. This is addressed in the text.

33. Line 323ff: Again tuberculosis needs to be mentioned.

Please see response to comment 6.

34. Sections: line 355ff, line 371ff, line 382ff and line 393ff, and line 407ff: have many parts in which discussion of respiratory disease in general is made but without the perspectives of tobacco consumption. In my opinion when not really linked to results of the paper not involving tobacco consumption this is not relevant for the current paper

Please see response to Comment 5.

35. Lines 432-434: Or the fact that general health improves and life expectancy increase and people live longer with respiratory disease and develop more bone changes.

Please see response to Comment 30.

36. Lines 456-460: therefore suggest leaving out some of these potential factors to get more clear picture of the impact of tobacco consumption.

Please see response to comment 5.

37. Line 488-489: Or they lived long enough to develop bone changes.

Please see response to Comment 30.

**Reviewer #5:** The submitted paper presents an application of biochemical data to palaeopathological explorations of respiratory health with consideration of whether tobacco consumption affects the prevalence of osteological evidence for respiratory disease. The results are interesting and highlight the complexities of identifying and adequately accounting for increasing environmental risk factors for disease that occurred with intensive industrialisation and urbanisation and how these can mask other factors that may present clearer data in other settings.
A number of comments are presented below which I think would improve the presented work in its clarity for readers.

Abstract
1- Page 1, Line 16: Typo identified – original text “Individuals were assed”. Assume this is meant to say, “Individuals were assessed”.

This has been corrected.

Main Text

2- Page 2, Line 44: Original text “While tobacco-use occurred frequently across all strata of society, the way people consumed became”. Suggested change “the way people consumed tobacco”. The sentence will read easier with it being explicitly being stated what people were consuming.

This sentence has been adjusted.

3- Page 3, Lines 54 – 62: Original text: “It induces respiratory inflammation, damages the tissues of the respiratory tract ….. oral diseases (23-26), to name but a few conditions associated with consumption of the drug.” This section has a repetitive feel to it due to the first two sentences both stating that the consumption of tobacco will increase infections/infectious diseases.
The opening sentence of this section of text could be improved by either a) removing the reference to infection (“promotes infection”) or b) moving this towards the end of the sentence and mentioning non-infectious disease too (“tract, increasing susceptibility to a range of infectious and non-infectious respiratory diseases”.
The start of the second sentence should also be reworked to remove “increases the risk of” portion as this will help reduce the sense of repetitiveness that currently exists across the first three sentences (lots of use of “increase”/”increased”).

This section has been improved according to your suggestions.

4- Page 4, Line 76: Original Text “Inskip and colleagues (32). There is inconsistent use of “and colleagues” versus “et al.” (used on line 79) through the paper.

This has been corrected.

5- Page 6, Figure 2, Caption: The reference for this figure (Davies-Barrett et al., in review) is not listed in the references at the end of the paper.

This paper is now fully published and has been added to the references.

6- Page 6, Line 139: Original text “‘rate payers’ (those who owned property)”. Please define rate payers in line 134 instead as that is the first use of the term in the paper.

This has been corrected.

7- Page 9, Lines 209 to 212: Original text “In binary logistic regression, if the upper limit of the 95% confidence interval falls below one”. The use of “one” (in text form) in this sentence and the sentence explaining the lower limit is unclear. Does the text mean that the 95% confidence intervals between groups are compared, or does the text mean that the limit of the falls above/below the numerical value of 1? If it is the latter, “one” should be written as “1” so it is clear that a numerical value is referred to here.

This has been corrected.

8- Page 9, Line 217 and Figure 4: Original text “However, a lower frequency of mature adults”. Frequency refers to the rate of something in a period of time or sample and so should not be used to refer to a number which is not a rate. Please change the use of “frequency” in this line of text to “number” and to “Number of individuals” on the y-axis of the figure.

This has been corrected.

9- Page 14, Figure 5: This is a really interesting way of visually representing the collective results from the study. I wonder whether numerical labels on the circles would help with the readability of the figure e.g. if 0°-180° is vertical with 0° at the top of the circle, 0° would have 0%, 90° and 270° would have 50% and 180° (bottom of the circle) would have 100%).

This is a very excellent suggestion. We have added this into figure 5.

10- Page 20, Line 408: Original text “(although not this result was)”. Assumed typo and “not” is meant to be “note”.

This has been corrected.
