# Supplementary material for: “A custome lothsome”: Investigating the association between tobacco consumption and respiratory inflammation in two post-medieval English populations (c. CE 1500–1855)
Source: PLoS One. 2025 May 22;20(5):e0324045. doi: 10.1371/journal.pone.0324045 (PMC12097713; doi:10.1371/journal.pone.0324045)
Supplement: Attachment — Submitted filename: V3 Comments to Reviewers.docx [file pone.0324045.s006.docx]

Authors’ responses to reviewers’ comments

We thank all reviewers for taking the time again to respond to the corrections made on our manuscript. We are very happy to hear that the manuscript is now considered of publishable quality. We include here our responses to the minor changes suggested by Reviewers 3 and 5.

Reviewer #3: I appreciate the efforts of the authors in preparing the revised version of the manuscript. The authors have addressed all comments and issues raised during the review process, resulting in significant improvements to the manuscript.

Although the issue of the limited sample size remains unresolved, it has been appropriately acknowledged and discussed in the Limitations section. In addition, the manuscript has been strengthened with thoughtful suggestions for future research.

This study provides a valuable contribution to the ongoing discourse on respiratory diseases and their potential association with tobacco consumption. In my opinion, the work by Davies-Barrett et al. is suitable for consideration for publication in PLOS One.

However, prior to publication, the manuscript should undergo careful language and editorial review. For example:
• Line 450, p. 22: Insert appropriate spacing around 10 µm, which is currently lacking.
• Line 478, p. 23: Amend the punctuation in “… deaths for untreated pneumococcal pneumonia occurred within six to ten days [95], Additionally, …” – a full stop should be placed after the citation: [95]. Additionally,
• Lines 622 (p. 28) and 624 (p. 29): In “7.4 – 36.7%” and “2.4 – 25.0%”, remove the surrounding spaces around the en dash.
• Line 638, p. 29: Remove the outer parentheses in ([80,82,84]) if already enclosed in square brackets, as per the citation style (i.e., should appear as [80,82,84]).

We have now undertaken careful proofing of the manuscript and have implemented all suggested changes provided.

Reviewer #5: Thank you to the authors for revising their manuscript and providing detailed responses to the points raised by the reviewers. I am happy with the changes that have been made both regarding the comments I raised, and those my fellow reviewers raised, and I would be happy to see the article published in its current state.
I would suggest one minor edit to the authors, but this is not an impediment to publication.
I feel the repetition of stating that analysis took place in the Human Osteology Lab at Leicester (lines 144/145 and 167/168) could be streamlined into a single line stating that “all analysis occurred in the Human Osteology Lab…..”. Whilst I personally do not think they are required information, I can understand that in the context of stating repository and curatorship information (journal requirement 3) of the human remains, it makes sense to highlight that analysis did not occur in the storage locations. But it would be simpler to state the analytical location once rather than repeating it.

We have implemented the suggested change.
